# Paraneoplastic dermatomyositis associated with urothelial cancer: report of a case and systematic review of the literature

**DOI:** 10.3389/fonc.2023.1223627

**Published:** 2023-10-31

**Authors:** Josep Sabaté-Ortega, Elisabet Bujons-Buscarons, Clàudia Fina-Planas, Núria Vilanova-Anducas, Noemí Vidal-Sarró, Núria Sala-González

**Affiliations:** ^1^ Oncology Department, Catalan Institute of Oncology, Hospital Universitari Dr. Josep Trueta, Girona, Spain; ^2^ Internal Medicine Department, Hospital Universitari Dr. Josep Trueta, Girona, Spain; ^3^ Department of Pathology, Hospital Universitari de Bellvitge.L’Hospitalet, Barcelona, Spain

**Keywords:** bladder cancer, polymyositis, dermatomyositis, urothelial cancer, dermato/polymyositis

## Abstract

**Background:**

The idiopathic inflammatory myopathies (IIM) are a collection of autoimmune diseases that have a substantial impact on the entire body and include conditions such as dermatomyositis (DM), polymyositis (PM), sporadic inclusion body myositis, and immune-mediated necrotizing myopathy. These disorders are characterized by symptoms such as muscular weakness, pain, and dermal rash. This systematic review is intended to explore the potential link between bladder cancer and DM/PM.

**Methods:**

We performed a comprehensive systematic search on PubMed and Scopus until August 2022 to identify relevant research studies. The studies that met our inclusion criteria focused on patients with urinary bladder cancer and dermatomyositis, and/or polymyositis.

**Results:**

The patients’ median age was 65.5 years (47–79), with the majority being male (15, 39.47%). Bladder cancer manifested before PM/DM in 5 (13.15%) patients, while in the majority of cases occurred after the cancer diagnosis. The stage of cancer at the time of the initial PM/DM diagnosis were mostly locally (11/20, 50%).During the first presentation, the patients had a median creatine kinase level of 2227 U/L, ranging between 44 and 10471. In one case, anti-TIF-1γ antibodies were found to be present. Among the cases with reported medical history (20/38), treatment immediately improved DM symptoms in 16 patients(53.8%) and in 3 patients(15%), symptoms of DM resurfaced during the period after the operation. Death was reported in 14 (36.8%) patients.

**Conclusion:**

In conclusion, our study provides knowledge and understanding for identifying specific risk factors in patients with the coexistence of bladder cancer and DM/PM and their management. During the initial and follow-up screening, age, gender, and the clinicopathological subgroup of myositis should be considered to ensure proper management of the condition.

## Introduction

The idiopathic inflammatory myopathies (IIM) constitute a diverse group of systemic autoimmune diseases, which include various subtypes such as immune-mediated necrotizing myopathy, sporadic inclusion body myositis, polymyositis (PM), dermatomyositis (DM) (1). The estimated prevalence of PM and DM ranges from 5 to 22 per 100,000 people, while the incidence rate among individuals at risk is approximately 1.2 to 19 million per year. The incidence rate of myositis is increasing over time due to enhanced detection rates. DM primarily affects individuals of two age groups, i.e., 5-15 years and 45-60 years. Conversely, PM rarely affects children, and the typical age of onset is between 50 to 60 years. The incidence rate of myositis among women is higher than that among men, with a ratio of 2 to 3:1 (2, 3).

DM presents mainly in heliotrope erythema, Gottron’s papules, telangiectasias, and other dermatological manifestations. Polymyositis (PM) is defined by symmetrical muscle weakness, elevated levels of muscle-derived enzymes, and is diagnosed through histopathological and electromyographic findings (4). The association between malignancy and DM is well known, and a coexisting malignancy is present in 20-25% of DM cases (5). Compared to PM, DM has a relatively higher risk of malignancy, being higher in men than in women (6). The cancers that exhibit strong association with DM are lung, ovarian, gastric, pancreatic cancer, colorectal, and non-Hodgkin’s lymphoma. Patients with PM have an increased risk of lung cancer, bladder cancer and non-Hodgkin’s lymphoma. While paraneoplastic myositis typically appears concurrently with underlying cancer, it can also manifest before or after the cancer diagnosis (7, 8).

The skin disorders caused by paraneoplastic dermatoses are varied and do not originate directly from cancer. Instead, they arise due to immune-mediated mechanisms or soluble factors that develop due to the immune response to the malignancy. This is why they can occur in regions far removed from the primary tumor site (9, 10). It has been speculated that elevated tumor antigens, which share similarities with muscle antigens, may trigger myositis by the antitumor immune response. On the other hand, other biomarkers, such as myositis-specific antibodies (MSA), are also related to a higher risk of malignancy. The most common MSAs associated with paraneoplastic dermatoses, although they also occur in idiopathic forms, are anti-transcriptional intermediate factor 1-gamma (TIF1), anti3-hydroxy -3-methylglutaryl-coenzyme A reductase (HMGCR), antinuclear matrix protein-2 (NXP-2) and autoantibodies against small anti-ubiquitin modifying enzyme (SAE) (1). For clinically atypical DM, these antibodies are substantial in the diagnostic procedure. They have a prognostic impact and can influence the therapeutic strategy. Despite this, their positivity occurs in 20-50% of DM cases, and the sensitivity and specificity of the available tests are highly variable, so their clinical applicability is limited (11).

The treatment of preneoplastic DM involves treating DM and treating malignancy, and its main objectives are to increase muscle strength and improve extramuscular manifestations (12). Treatment of malignancy is often insufficient to control myopathic and cutaneous manifestations of associated DM (13). Systemic corticosteroids, at a dose of 0.5-1.0mg/kg/day for at least one month with gradual dose reduction, are considered the optimal treatment in 1aL. Despite this, long-term treatment (6-12 months) is often necessary, so the introduction of steroid-sparing immunosuppressive treatment can prevent the development of serious side effects related to prolonged use of steroids.

Growing evidence suggests that patients with idiopathic inflammatory myopathy/dermatomyositis (IIM/DM) may share a similar risk of comorbidities as systemic lupus erythematosus (SLE) patients, with antimalarials like hydroxychloroquine (HCQ) are considered as effective and safe options for DM, albeit with varying response rates and potential risks of exacerbating skin disease or causing idiosyncratic skin hypersensitivity reactions. Studies indicate that intravenous immunoglobulins may be an effective option for patients resistant to traditional therapies (5).

In this article, we report a case of paraneoplastic PM in patient with a muscle invasive urothelial carcinoma distant relapse. Likewise, we carried out a systematic review of the literature to determine the relationship between urothelial cancer of the bladder and urinary tract and DM or PM, with the ultimate goal of establishing urothelial cancer as one of the underlying causes of idiopathic inflammatory myopathies.

## Presentation of the case

**Figure 1:**
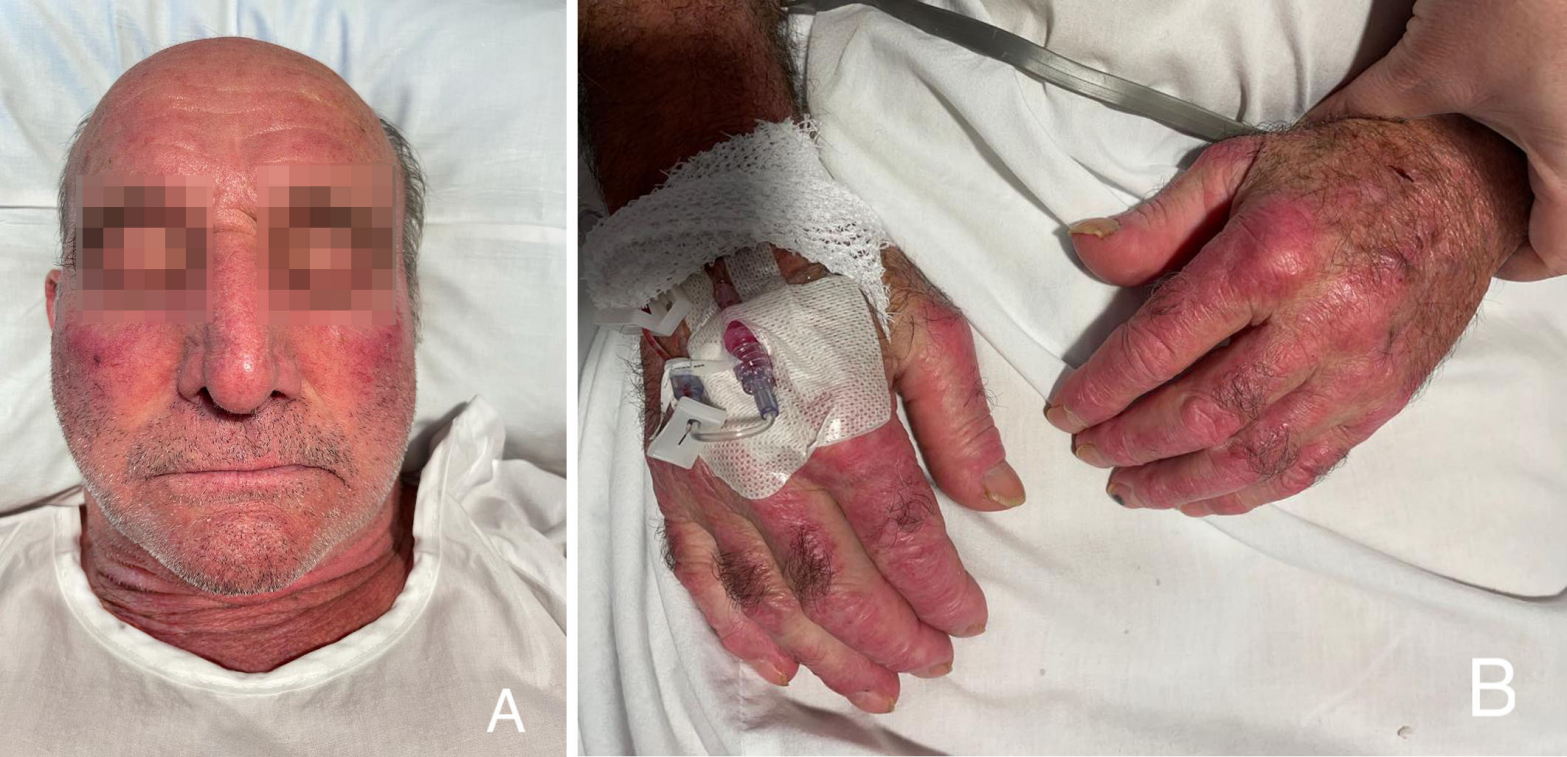
Clinical signs of dermatomyositis: **(A)** Gottron’spappules; **(B)** Heliotrope erythema.

**Figure 2:**
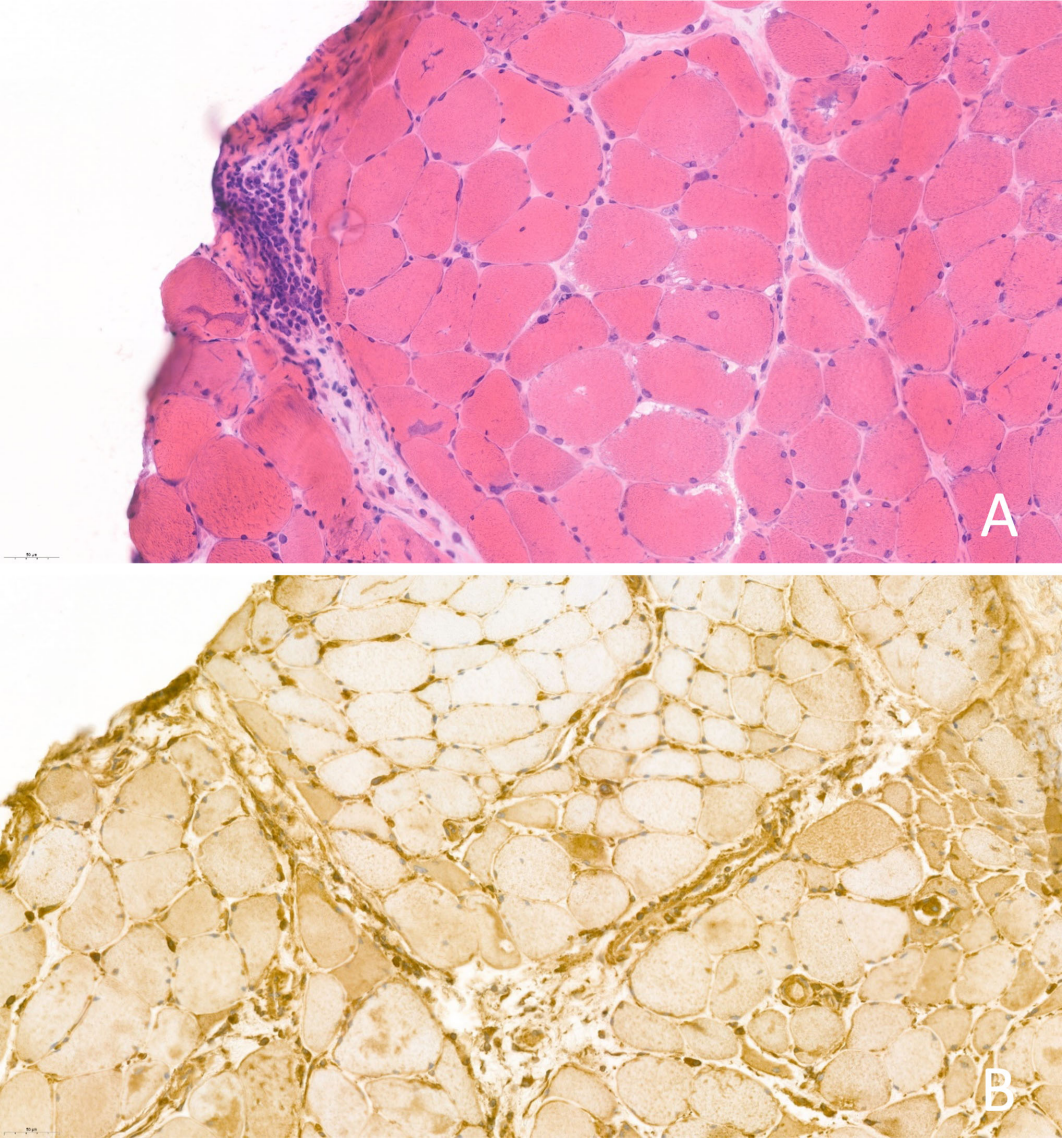
**(A)** Hematoxylin-eosin staining 40x. Muscle biopsy with perifascicular atrophy and inflammatory cells; **(B)** MHC class I immunostaining 40x.

A 75-year-old patient with a history of locally advanced high-grade urothelial carcinoma underwent radical cystectomy, lymphadenectomy, and Bricker-type cutaneous ureteral ileostomy in December 2020, not considering himself a candidate for adjuvant treatment with platinum in a context given a history of severe hearing loss and peripheral claudication. Due to dysphagia to solids and liquids, erythematous-violaceous lesions in the joints (**Figure 1**), pain in the upper extremities and proximal and symmetric weakness of the upper extremities with a CK elevation >500 and significant underlying functional limitation of a month and a half evolution, he was admitted for study in November 2021 with a diagnosis of probable dermatomyositis under study. Treatment with bolus corticosteroids was started on admission, escalating treatment to gamma globulins and methotrexate, given the clinical severity. During hospital admission, electromyography and magnetic resonance imaging were performed with findings compatible with diffuse myopathy and inflammatory signs and confirmatory muscle biopsy (**Figure 2**) with weak anti-TIF-1γ positivity, suggesting a paraneoplastic etiology. A PET-CT confirmed hypermetabolic 7mm adenopathy in the right internal iliac chain, suspicious of tumor recurrence. Chemo-radiotherapy treatment 30.6Gy was started in 34 concurrent sessions with weekly carboplatin during January and February 2021 with a complete response. After completing the treatment with chemo-radiotherapy, the patient presented a favorable evolution of the debut symptoms, with a complete resolution of muscle weakness and dysphagia after a few months.

## Materials and methods

### Search approach and eligibility criteria

We systematically searched Scopus and PubMed up to August 2022 using the following keywords: (urinary bladder neoplasms) and (polymyositis or myositis, or dermatomyositis). Two authors did the research independently. The inclusion parameters were precisely defined, and there were no abnormalities in the search results. We analyzed all the articles that examined the association between neoplasms of the urinary bladder and DM or PM; people that were diagnosed with malignant neoplasm of the urinary bladder and presented DM and/or PM.

The combination of terms that yielded the best results in both search engines was the following: (“Urinary Bladder Neoplasms”[MeSH Terms] OR “Urinary Bladder Neoplasms”[Text Word]) AND ((“Dermatomyositis”[Mesh] OR “Dermatomyositis”[Text Word]) OR (“Polymyositis”[Mesh] OR “Polymyositis”[Text Word]) OR (“Myositis”[Mesh] OR “Myositis”[Text Word])), other than English, Spanish, review articles, book chapters, editorials/letters/conference presentations, as well as animal studies or surveys. Our investigation did not include letters that did not mention any association between urinary bladder cancer and DM or vice versa. In the systematic review, the Preferred Reporting Items for Systematic Reviews and Meta-Analyses (PRISMA) standards were followed (14).

### Data screening

Initially, the authors independently screened the data, removing duplicate records using EndNote X9 software30. Articles of interest were screened for eligibility by reviewing the titles and abstracts. If the information was insufficient in the title and abstract, papers were further assessed by screening the full text. **Figure 1** outlines the study selection procedures.

### Data retrieval and verification

The reviewers independently extracted data from individual studies and presented it in standardized forms (**Table 1**), elaborating on the information extracted from the included original articles (epidemiological studies), reviews, letters to the editor, and case reports. The data extracted included the year of the study conducted, gender and several observed cases, initial and secondary diagnosis, symptoms and laboratory findings, treatment and management of cancer and DM/PM, outcomes and results of individual studies. Median age and laboratory findings were calculated, while descriptive analysis (i.e., frequency) was used for other continuous variables. We utilized the NIH Quality Assessment tool to analyze the quality of the included studies (**Table 2**).

**Table 1:** Clinical management and outcome among reported cases of Bladder cancer and paraneoplastic myositis/DM.

Reference	Year	Study type	Age (Yrs)/Gender	Cancer Type	Staging	Initial Diagnosis	Secondary Diagnosis	Laboratory Findings	Signs and Symptoms	Treatment for Cancer	Treatment for IIM	Outcome of Treatment
W. M. H. Behan et al.	1982	CR	69 M	TCC	Local	PM	BC	AST 72 IU/L, ALT 901 U/L, CPK 3800 U/L	Weakness, slurred speech, difficulty in swallowing, itching, rash on abdomen and whole body, bilateral ptosis, left lateral rectus muscles weakness, dysarthria, dysphagia, muscle weakness (limbs) and Hashimoto’s thyroiditis.	Tumor resection	Mestinon ACTH and hydrocortisone lotion	Initially respiratory arrest despite mestinon, Osteoporosis due to steroid
Almog Y et al.	1991	CR	76 M	TCC	Local (recurrence)	BC	DM	NA	NA	NA	Steroids (1mg/kg) and azathioprine	Kaposi’s sarcoma (skin) Discontinuation of treatment resulted in significant improvement
Marchal Escalona C et al.	1992	CR	–	TCC	–	DM	BC	NA	Erythematous (face and upper body), heliotrope erythema (periorbital and fingernails) and telangiectasia	NA	NA	NA
Michael L. Jensen et al.	1997	CR	64 M	TCC	Local	PM	BC	CPK 1790 U/L, LDH 428 U/L.	Weakness, fatigue, cough and severe hypotension	Radical cystoprostatectomy with ileal loop urinary diversion	Steroids (1mg/kg)	Increased functional strength (attained independence of daily activity)
Constantine B et al.	1997	CR	73 F	TCC	Local	PM	BC	CPK 3540 IU/L, LDH 412 IU/L, AST 163 IU/L and ALT 178 IU/L	Dysphagia, muscle weakness (limb and shoulders) and decreased strength	Tumor resection and RT	NA	Muscular symptoms improved, and enzymes returned to normal after treatment.
E. Mallon et al.	1999	CR	75 M	TCC	Local	DM	BC	CPK 7650 U/L	Rash and erythema (face and upper body), periorbital oedema, Gottron’s papules, telangiectasia, muscle weakness	Tumor resection, RT, Intravenous gammaglobulins (2 g/kg), CT- cyclophosphamide and vincristine (3 courses)	Steroids (1mg/kg)	Muscular strength and cutaneous symptoms improved
N.Y.Talanin et al.	1999	CR	62 M	TCC	Metastatic	BC	DM	Positive RPR: 1:4, ANA titer 1:80, Anti smooth muscle antibody titer 1:40.	Erythema (face and whole body), swelling (face), haematuria, weight loss, with periorbital edema and muscle weakness	Radical cystoprostatectomy with ileal loop urinary diversion, Pelvic RT	Triamcinolone 0.1%	Improved in oedema and erythema
Russ BW, et al.	1999	CR	–	TCC	–	DM	BC	NA	NA	NA	NA	NA
Federman DG et al.	2000	CR	68 M	TCC	Local	BC	DM	CPK 815 U/L	Muscular pain and weakness, Gottron’s papules, and photosensitive erythema (face with induration)	Tumor resection	Steroids (1mg/kg)	Dermatomyositis (DM) resolved for 12 months
A. J. Robinson et al.	2001	CR	61 M	Poorly diferenciated TCC	Metastatic	BC	DM	CPK 8925 U/L, AST 323 U/L	Micturition and haematuria, malaise, muscular pain, skin rash, erythematous, heliotrope erythema (periorbital)	Tumor resection and RT	Steroids (1mg/kg) and azathioprine.	Clinical condition improved, and enzymes returned to normal after treatment
Hideaki Sekineet al.	2002	CR	47 F	Adenocarcinoma	Metastatic	BC	DM	CPK937 U/L, LDH1027U/L, Carcinoembryonic antigen 46.3 ng/ml	Erythema (face and upper body), swelling on the face with oedema	Tumor resection, RT and CT (methotrexate, vinblastine, doxorubicin and cisplatin (2 courses))	Steroids (1mg/kg)	Skin and muscle symptoms improved
R Apaydinet al	2002	Letter to Editor	63 M	TCC	Local	DM	BC	CPK 44 U/L	Skin rash (face, upper body and joints), heliotrope erythema, oedema (eyelids), telangiectasia (face and chest) and Gottron’s papules	NA	Steroids (1mg/kg)	Skin lesions did not improve markedly
William R. Rankin et al	2002	CR	64 M	TCC	Locally advanced	DM	BC	Cytokeratine 500UI/l	NA	Tumor resection and CT- gemcitabine and paclitaxel	NA	Enzymes returned to normal after treatment
Isao Takeda et al.	2004	CR	56 M	Adenosquamous cell carcinoma	Metastatic	PM	BC	CPK 5298 IU/L, AST 206 IU/L, ALT 165 IU/L, LDH 1763, and myoglobin-5334 mg/dl	Muscular weakness, dysphagia and weight loss	NA	Steroids (bolus), methotrexate 6mg and cyclosporin(100mg)	Overall condition improved
A. Hafejeeet al.	2004	CR	75 M	TCC	Local	DM	BC	CPK 1237 IU/L	Muscular weakness (arms and legs), dysphagia, peristalsis impairment	NA	Steroids (1mg/kg) and IVIG (2g/kg)	Condition improved after steroid and IVIG, and enzymes returned to normal after treatment
LiorSagiet al.	2009	CR	72 M	Small cell carcinoma of the bladder	Metastatic	DM	BC	AST 475 IU/L, LDH 760 IU/L, CPK 10471IU/L, fructose-bisphosphate aldolase 19.1 IU/L, ANA 1:40	Erythema (scalp and face), periorbital swelling, poikiloderma (chest), fever, telangiectasias, hemorrhagic bullae (fingers and palms), papules (joints) petechia (upper palate and lips)	NA	Steroids (1mg/kg), IVIG 25g	Moderate improvement
Ran Xu et al.	2013	CR	60 M	TCC (Grade III)	Local	BC	DM	CPK 583.9U/L, LDH 578 U/L, myoglobin 1052.6 ug/L.	Muscular weakness	Radical cystoprostatectomy	Steroids (1mg/kg)	Overall condition improved
Xiangdong Liu et al.	2013	Original article	18	Urological cancer	NA	DM	BC	NA	NA	NA	NA	Immunosuppressive treatment for certain autoimmune diseases may promote tumor progression
John Fitzpatrick et al.	2014	CR	67 F	TCC	Local and metastatic relapse	DM	BC	NA	NA	Tumor resection and RT and CT at recurrence	NA	NA
RiccardoBientinesiet al.	2016	CR and Literature Review	79 M	TCC (carcinoma in situ)	Locally advanced and metastatic relapse	BC	Paraneoplastic DM	CPK 2227 UI/L, LDH 636 UI/L	Muscular pain and weakness, dysphagia, dysphonia, and erythematous (face, neck and back)	Tumor resection	Steroids (1mg/kg) and azathioprine (150 mg/day)	Poor response
Emilio Sacco et al.	2009	Review	NA	Genitourinary tumors	NA	BC	Paraneoplastic DM	NA	1. PNHC: Cushing’s syndrome, weight gain, sweating, telangiectasia, red striae, proximal muscle weakness, Osteoporosis, hyperpigmentation and lipodystrophy, persistent hypertension, insulin resistance and psychological disturbances. 2. Muscle weakness (results from hypokalemia) 3. RCC with PNS: Fever, anaemia, weight loss, and fatigue 4. HHM with PNS: polymorphic, specific symptoms including asthenia, headache, lack of appetite, nausea, vomiting, constipation, polyuria polydipsia, acute confusional or lethargic state, coma-associated hypercalcemia	Radical treatment of tumor or a neoplastic debulking, Palliative treatment.	NA	NA
José Mario Sabio	2006	Letter to Editor	79 M	TCC	Local	DM	BC	CPK 2227 UI/L, LDH 636 UI/L,	Benign prostatic hyperplasia, erythematous rash (face and upper body), heliotrope erythema and lichenoid papules, muscular pain and weakness, dysphagia and dysphonia,	Tumor resection	Steroids (1mg/kg) and azathioprine (150 mg/day)	Overall condition improved, but dysphagia persisted

CR, Case report; TCC, Transitional cell carcinoma of the bladder; PM, Polymyositis; BC, Bladder cancer; AST, Aspartate transferase; ALT, Alanine aminotransferase; CPK, Creatine phosphokinase; ACTH, Adrenocorticotropic hormone; DM, Dermatomyositis; LDH, Lactate dehydrogenase; RT, Radiotherapy; RPR, Rapid plasma regain; ANA, Antinuclear antibody; PNS, Paraneoplastic syndromes; PNHC, Paraneoplastic hypercortisolism; RCC, Renal cell carcinoma; Humoral hypercalcemia of malignancy, Intravenous immunoglobulins; NA, Not available.

**Table 2:** Quality Analysis of Eligible Studies.

First Author (Year)	NIH Quality Assessment Tool for Case report Studies									Quality (Total Quality Score)
	Q1	Q2	Q3	Q4	Q5	Q6	Q7	Q8	Q9	
W. M. H. Behan et. Al.1982 (15)	*	*	NA	*	*	*	*	*	*	Good (8)
Almog Y et. al.1991 (16)	*	*	NA	*	*	*	*	NA	*	Good (7)
Marchal Escalona C et. Al.1992 (17)	*	*	NA	*	*	*	*	NA	*	Good (7)
Michael L. Jensen et. Al.1997 (18)	*	*	NA	*	*	*	*	*	*	Good (8)
Constantine Bouropoulos et al., 1998 (19)	*	*	NA	*	*	*	*	NA	*	Good (7)
E. Mallon et. Al. 1999 (20)	*	*	NA	*	*	*	*	NA	*	Good (7)
N.Y.Talanin et. Al.1999 (21)	*	*	NA	*	*	*	*	NA	*	Good (7)
Russ BW, et.al. 1999 (22)	*	*	NA	*	*	*	*	NA	*	Good (7)
Federman DG et.al.2000 (23)	*	*	NA	*	*	*	*	NA	*	Good (7)
A. J. Robinson et.al.2001 (24)	*	*	NA	*	*	*	*	NA	*	Good (7)
Hideaki Sekine et al.2002 (25)	*	*	NA	*	*	*	*	NA	*	Good (7)
R Apaydin et. Al.2002 (26)	*	*	NA	*	*	*	*	NA	*	Good (7)
William R. Rankin 2002 (27)	*	*	NA	*	*	*	*	NA	*	Good (7)
Isao Takeda et al., 2004 (28)	*	*	NA	*	*	*	*	NA	*	Good (7)
A. Hafejee et.al.2005 (29)	*	*	NA	*	*	*	*	NA	*	Good (7)
LiorSagi et.al.2009 (30)	*	*	NA	*	*	*	*	NA	*	Good (7)
Ran Xu et. al.2013 (31)	*	*	NA	*	*	*	*	NA	*	Good (7)
Xiangdong Liu et al., 2013 (32)	*	*	*	*	NA	*	*	*	*	Good (8)
John Fitzpatrick et.al.2014 (33)	*	*	NA	*	*	*	*	NA	*	Good (7)
Riccardo Bientinesi et.al.2016 (34)	*	*	NA	*	*	*	*	NA	*	Good (7)
Emilio Sacco et al.2009 (35)	*	*	NA	*	*	*	NA	NA	*	Poor (3)
José Mario Sabio 2006 (36)	*	*	NA	*	*	*	*	NA	*	Good (7)

Q1: Was study question or objective clearly stated?, Q2: Was study population clearly and fully described, including case definition?, Q3: Were cases consecutive?, Q4: Were subjects comparable?, Q5: Was intervention clearly described?, Q6: Were outcome measures clearly defined, valid, reliable, and implemented consistently across all study participants?, Q7: Was length of follow-up adequate?, Q8: Were statistical methods well-described?, Q9: Were results well-described?, Good: Met 7–9 criteria, Fair: Met 4–6 criteria, Poor: Met 0–3 criteria. NA, not applicable; NIH, National Institutes of Health; NR, not report.

## Results

**Figure 3:**
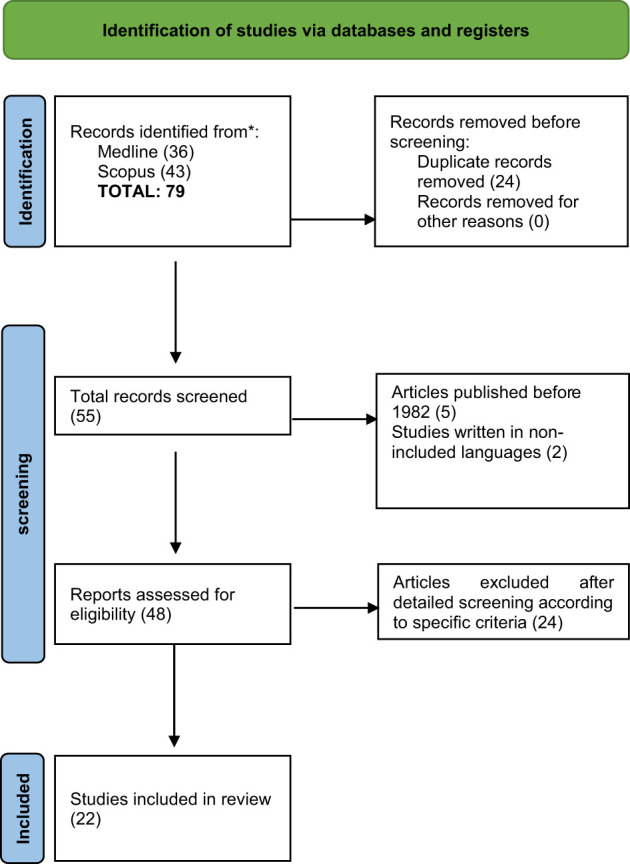
PRISMA flow chart.

A total of 79 articles were retrieved from in-dept screening of electronic databases (PubMed, Pubmed Central and Scopus). After removing duplicates (n= 24), 55 records were retained, 7 were excluded by reviewing the titles or abstracts, and 48 full texts were reviewed. Subsequently, these articles were screened against the eligibility criteria, and 22 studies, including 18 case reports, one original article (epidemiological study), one review and two letters to the editor (description of a case) were included in our review (**Figure 1**). The 22 included were published from 1982 to 2016 (**Figure 3**). The median age of the patients was 67.5 years (range 47–79 years), the majority of which were males 15/38(39.47%) and females 3/38 (7.89%). The status of gender for other patients was unknown. Based on the available medical literature, the medical history was reported in 20 out of 38 cases (case reports & letter to editors).The type of cancer reported among the selected cases included transitional cell carcinoma in 17/20 (85%), which was the most frequent histological type, adenocarcinoma, adenosquamous carcinoma and small cell carcinoma of the bladder in 1/20 (5%) patients, respectively. The stages of cancer at the time of the initial diagnosis were referred in 18/20 (90%) patients; 11/20 (50%) locally, 2/20 locally advanced and 5/20 metastatic. Bladder cancer manifested before DM in 5/38 (13.15%). Creatine kinase was a median level of 2227 U/L (range 44–10471) reported at the time of the first presentation of symptoms. Out of 20 patients, 14 were tested for antibodies, 11/14 (78,5%) were negative, 3/14 (21,4%) were positive for ANA (Antinuclear Antibody), while anti-Ro, anti-smooth muscle, anti-thyroglobulin and anti-microsomal antibodies were reported in 1/14 (7,1%) patient each, respectively. Immediate improvement in the symptoms of DM was observed after treatment in 16/20 (80%) patients. Regarding the type of surgical treatment performed, the tumor was resected in 14/20 (70%) patients. Cancer recurrence occurred in 6/20 (30%). Fatalities were observed in 14/38 (36.8%) patients (7/14 (50%) with complications including multi-organ dysfunction and infection, post-operative deep vein thrombosis and small bowel perforation, respiration insufficiency, pneumonia and sepsis) while 2/38 (5.26%) patients reported improved condition during long-term follow-up.

## Discussion

Paraneoplastic dermatomyositis is a rare autoimmune condition which serves as a potential early warning sign of an undiagnosed cancer. Typical changes to the skin include a heliotrope rash on the eyelids and an erythematous rash on the joints, such as Gottron’s papules. Progressive, symmetric proximal muscle weakness and dysphagia are classic manifestations specific to the disease upon examination of the patient (37). Beside skin changes, erythematous-violaceous lesions and muscle weakness, we observed increased levels of muscle-derived enzymes, including creatine phosphokinase (CK), lactate dehydrogenase (LDH), aldolase, aspartate aminotransferase (SGOT), and alanine aminotransferase (SGPT) (37). These reactions to muscle fibers and skin are caused by the release of bioactive molecules in the body (38, 39).

TIF1-γ functions as a tumor suppressor, mediator of DNA damage repair, transcription regulator, and E3 ligase modulates TGF-β signaling. In cancer-associated DM with TIF1-γ, there is a hypothesis that cancer might be the underlying cause of DM and that TIF1-γ could function as a tumor autoantigen (40, 41). In 2006, Targoff spotted an anti-TIF-1γ antibody (a protein of 155-kD) in DM patients. This protein involves various pathways, including transforming growth factors as agonists or antagonists. In 2010, Selva-O’Callaghan conducted a meta-analysis demonstrating that anti-TIF-1γ significantly increased the risk of malignancies by 18-fold. Previous studies have also highlighted the correlation between anti-TIF-1γ and malignancies in DM or PM (42). Conversely, in our analysis, only one case out of 22 selected studies reported the presence of anti-TIF-1γ.

Our study found that bladder cancer manifested before PM/DM in 5 (13.15%) patients, while in the majority of the cases, it occurred after the cancer diagnosis. Regarding the onset of the disease, mixed theories have been observed. Liu Y reported that 69.77% of malignancies occur before or after DM (43). Andras C reported that 64.8% of malignancies appear within one year after the diagnosis of DM (44). In contrast, Chang L reported that the diagnosis of malignancies occurred after one year of DM in 30 (46.15%) cases, with a 61.54% diagnosis rate within 3 years. Qiang JK identified 17.29% instances of DM in malignant patients, observed in 2 to 5 years in 2.7% of cases (6). The leading cause of death in non-cancer patients with PM is typically cardiac infarction. In DM patients with and without cancer, the most common cause of death is respiratory failure, particularly due to pneumonia and pneumonitis (45). Our study found that 2 DM patients died due to respiratory insufficiency and pneumonia, while 3 patients (2 with DM and 1 with PM) had a history of cardiac complications.

Hill categorized DM and PM based on distinct cancer types. DM-associated cancers encompass ovarian, lung, pancreatic, gastric, and colorectal cancers, whereas bladder, lung, and non-Hodgkin lymphoma are primarily linked with PM (46). Regarding risk factors, males are predominantly afflicted with DM and PM, despite higher incidence reported in females (47). Furthermore, aged individuals are more vulnerable in the context of these conditions. Stockton et al. reported that patients aged between 45 to 75 years have an increased risk of developing cancer following the onset of DM (48). Another study accentuated the 40 to 59 years age group and those over 80 years (49). In line with these findings, our research supports the idea that patients with cancer-associated myositis tend to be older (median age for cases was 67.5 years), with peaked incidences in male patients (39.47%).

Recognizing dermatomyositis in a patient can prompt further investigation and earlier cancer detection, improving the chances of successful treatment and prognosis. Therefore, it is essential for clinicians to thoroughly examine those with a newly diagnosed condition of DM to determine if it is complicated by malignant diseases, along with a review of the previous history of signs and symptoms (38, 45). Also, a comprehensive cancer screening should be performed within the first 3 to 5 years after the onset of the disease, although the optimal screening regimen for recently diagnosed DM patients has yet to be defined. Improvement analysis can be determined by combining the results of the physical examination with the observed restorations in muscle strength, reduced fatigue, and enhanced skin condition. Furthermore, physical examination of DM patients should be recommended for lifelong monitoring, especially during the initial 3 years after diagnosis (50).

Nevertheless, it is important to acknowledge the limitations of our review. Our study’s small number of patients underscores the rarity of the association between DM/PM and bladder cancer. The heterogeneity of data with missing variables in some studies may result in significant differences in latency periods, making it difficult to draw definitive conclusions. Moreover, a possible crossover effect of 2 or more treatments given to patients may make it challenging to attribute the response to a specific treatment. Nonetheless, this systematic review provides insight into the literature and advances our understanding of the clinical features and laboratory findings of patients with bladder cancer associated with myopathies.

## Conclusions

In conclusion, our study provides knowledge and understanding for recognizing specific risk factors in patients with the coexistence of bladder cancer and DM/PM and their management. Based on our findings and available literature, the identified risk factors are severe, intensified, or atypical cutaneous symptoms in older patients, rapidly progressing severe muscle weakness, and persistently elevated serum muscle enzymes. Therefore, proper screening of individual patients is recommended, based on the clinicopathological subgroup of myositis, age, and gender, and this awareness should also extend throughout the follow-up. It is essential to check for relapse in patients with increased symptoms of the autoimmune condition, as symptoms occasionally appear after the tumor resection. Future studies on a large scale with multicentered data are needed to further characterize the clinical features of these conditions.

## Author contributions

JS-O conducted the review and wrote the manuscript with input from all authors. EB-B and CF-P contributed to conduct the review. NS supervised the findings of this work. All authors contributed to the article and approved the submitted version.
